# Policy Guidelines for Effective Inclusion and Reintegration of People with Chronic Diseases in the Workplace: National and European Perspectives

**DOI:** 10.3390/ijerph15030493

**Published:** 2018-03-11

**Authors:** Anastasia Vlachou, Panayiota Stavroussi, Olga Roka, Evdokia Vasilou, Dimitra Papadimitriou, Chiara Scaratti, Asel Kadyrbaeva, Klemens Fheodoroff, Valentina Brecelj, Olga Svestkova, Beata Tobiasz-Adamczyk, Jon Erik Finnvold, Sonja Gruber, Matilde Leonardi

**Affiliations:** 1 Department of Special Education, University of Thessaly, Argonafton-Filellinon Str., 38221 Volos, Greece; stavrusi@uth.gr (P.S.); olgaroc1@yahoo.gr (O.R.); eudokia1@hotmail.com (E.V.); dpapad@upatras.gr (D.P.); 2 Neurology, Public Health and Disability Unit, Foundation IRCCS Neurological Institute “Carlo Besta”, Via Celoria 11, 20133 Milan, Italy; Chiara.Scaratti@istituto-besta.it (C.S.); Matilde.Leonardi@istituto-besta.it (M.L.); 3 European Association of Service Providers for Persons with Disabilities (EASPD), Rue du Commerce 72, 1040 Brussels, Belgium; Asel.Kadyrbaeva@easpd.eu; 4 Gailtal Klinik—Neurologische Rehabilitation, 9620 Hermagor, Austria; klemens.fheodoroff@me.com; 5 Development Centre for Vocational Rehabilitation, University Rehabilitation Institute, Ljubljana 1000, Slovenia; valentina.brecelj@ir-rs.si; 6 Department of Rehabilitation Medicine, 1st Faculty of Medicine Charles University and Vseobecna Fakultni Nemocnice V Praze, 12808 Praha, Czech Republic; olga.svestkova@lf1.cuni.cz; 7 Department of Epidemiology and Preventive Medicine, Jagiellonian University Medical College, 31-008 Krakow, Poland; mytobias@cyf-kr.edu.pl; 8 Norwegian Social Research, OsloMet—Oslo Metropolitan University, Postboks 4 St. Olavs plass, 0130 Oslo, Norway; jon.e.finnvold@nova.hioa.no; 9 Department of Disability and Diversity Studies, Carinthia University of Applied Sciences, 9020 Klagenfurt, Austria; S.Gruber@fh-kaernten.at

**Keywords:** chronic diseases, perspectives, national stakeholders, European stakeholders, policy guidelines, work re-integration, work inclusion

## Abstract

The increasing prevalence of chronic diseases among the European working age population, as well as the implications for the individual and societal level, underline the need for policy guidelines targeting the effective inclusion of persons with chronic diseases in the workplace. The aim of the present paper was to explore the perspectives of European and National-level stakeholders on existing strategies for work re-integration of persons with chronic diseases, and to provide policy guidelines. A highly-structured interview protocol was distributed to 58 National level stakeholders (policy makers, professionals and employers) from seven European countries. Additionally, 20 European organizations concerned with health-related issues and employment completed an online survey. The findings reveal that employment-related challenges remain largely unaddressed. Both national and European stakeholders considered the existing legislative frameworks inadequate and appraised the co-ordination for the implementation of employment re-integration policies as ineffective. Policies targeting at work re-integration of persons with chronic diseases at European and national level should focus on consistent cooperation among all key stakeholders, awareness raising to staff and management, dissemination of effective strategies, developing research and evaluation standards and establishing monitoring systems on inclusive labour markets.

## 1. Introduction

Work and health are interrelated in many ways. The increasing prevalence of non-communicable diseases among the European working age population, combined with the dramatic low employment rates of persons with chronic diseases (PwCDs), is an indicative depiction of this particular relation. Recent data from 27 EU member states, showed that about one quarter of the working age population (23.5%) had a chronic disease, while 19% reported having long-standing health issues [1]. There is ample evidence indicating that chronic diseases: (a) endanger the health and well-being of people, leading them to disability and premature death [1,2]; (b) expose individuals to economic deprivation, through unemployment and economic inactivity [3,4,5,6]; (c) are responsible for a huge loss of potentially productive life years of working age persons [7]; and (d) can become a risk factor for absolute or relative poverty and social exclusion [8]. According to the 2011 ad-hoc module of the EU Labour force survey [9], the employment rate of persons with long-standing health issues, in the EU Member States, was nearly 30% lower than the percentage of those without such issues; a significant gap that has become wider, during the post-recession years [10].

These data suggest that a significant number of working age European citizens are either excluded from the open labour market, facing long-term unemployment [11] or are economically inactive being/becoming recipients of scarce passive measures, such as disability benefits and pension [12]. From an economic point of view, this results in potential loss for the economy [13], major costs, and ever-increasing government spending on disability benefits [1,14]. From a socio- political approach, workers with longstanding health problems are placed in a precarious position, between an increasingly hostile welfare state (for an extensive analysis see: [15]) and a labour market in which the “able-body/mind” remains a largely unquestioned norm. In either case, PwCDs are in a particularly unfavorable position in Europe’s labour market.

Within this context, a growing interest on employment for PwCDs has been developed with paid work, positioned as the principal mechanism to secure social inclusion [15]. In many Western countries, disability assistance programmes have been restructured to encourage paid work, while in different regions (i.e., U.S., Australia, and EU) active labour market procedures have been developed to increase social and economic participation of persons with disabilities and/or chronic diseases. Further, in most EU member states, PwCDs are included in provisions and programmes, made for persons with disabilities, like the “European Disability Strategy 2010–2020” [16], aimed at moving people from welfare to workfare systems. These actions have been partially influenced by broader movements, such as the International Disability Rights movement and more specifically the Convention on the Rights of Persons with Disabilities (CRPD), which aimed at promoting, protecting and ensuring the full and equal enjoyment of all human rights -including the right to work—by all persons with disabilities.

While the above “human-rights approach”, as well as the “employment-focused social inclusion strategies”, are of immense importance they still do not suffice for ensuring and securing inclusion in the open labour market [17]. Firstly, it is important to mention that, the right to work applies mainly to persons with disabilities and, even though the notion of disability is broadly defined so as to include PwCDs, in many countries, disability equals primarily to physical, sensory or intellectual impairments. Thus, from the point of view of the social security systems, it might be expected that chronic illness is not of primary interest, since it is not, of itself, grounds for receiving benefits. Instead, many systems define access in relation to some methods of calculating loss of function, especially in relation to the ability to work [18].

Secondly, in order for disability legislation to bring about change, it must be accompanied by efforts to develop effective and integrated strategies, that is policies, programmes/systems and services with a real positive impact on the lives of PwCDS [13,19,20]. In practice, however, many work re-integration programmes focus principally on normalizing the individual, employing experts and specialists to improve his/her human capital and employability. This focus has not been accompanied by strategies, focusing on creating contexts for employment that ensure accessibility and accommodation in particular, and job security and living wages more generally. When governments have made employers and working conditions a focus of policy, programmes have typically emphasized voluntary compliance and incentives, rather than mandatory changes [15]. Even more important is the fact that many initiatives, designed to re-integrate persons with disabilities and/or chronic diseases into the labour market, assume that there are sufficient numbers of jobs/employers out there for persons with disabilities/chronic diseases, wishing to move into paid employment, and that these opportunities provide liveable wages, secure employment relations and the necessary ‘flexibility’ to accommodate workers (for an extensive analysis see: [15,21]). On the contrary, the dominant characteristics of contemporary economy (i.e., globalization, transnational ownership, disinvestments, casualization and flexibility, rise of low-paid unskilled service employment) render the development of inclusive working environments a complex political, economic and social issue. 

Given the complexity of the situation, building a more inclusive labour market requires action of workers, employers and their representatives and public authorities, at both EU and national levels to ensure that obstacles can be identified and overcome [1]. At a micro level of analysis, it involves the person and his/her family that have to adjust to chronic diseases, the employer and colleagues, who constitute the context [3], but also medical professionals and experts, who are key actors on optimizing health outcomes [22]. At a macro level, the active inclusion of PwCDs in the open labour market is an exceptionally political and economic issue, as it deals with complex notions of social justice, equal opportunities, and social policy goals and it involves policy makers and economists [7,8,23]. Here, it is important to actively involve different and explore their views, concerning (a) the current situation (i.e., knowledge/availability/effectiveness) of strategies for work re-integration of PwCDs in Europe and across European countries, and (b) the opportunities to promote participation of the latter in the labour market. 

In light of the above, the present paper focuses on investigating the perspectives of different stakeholders, at both EU- and national-level(s), concerning the work re-integration of PwCDs. Study 1 (“national-level survey”) referred to national level stakeholders and aimed to explore their views on a. the level of implementation of particular strategies, relevant to employment re-integration of PwCDs at a national level, as well as b. the importance and the level of implementation of specific recommendations. Potential differences among different *types of stakeholders* and different *social welfare models* were also explored. Study 2 (“European-level survey”) explored the view and perspectives of European-level stakeholders on a. the level of implementation of particular strategies, relevant to employment re-integration of PwCDs at EU level, as well as b. the importance of specific recommendations. Suggested barriers and further recommendations were also explored. In combination, the results of this two-part study can function as a basis, upon which specific guidelines and policy recommendations, at National and EU Levels, for the work re-integration and inclusion of PwCDs can be developed. 

## 2. Materials and Methods 

### 2.1. Design

The two studies were carried out within the scope of the EU-funded Participation to Healthy Workplaces And Inclusive Strategies in the Work Sector (PATHWAYS) project. The final objective of this 3-year project is to develop guidelines to support the implementation of effective professional re-integration strategies for PwCDs. Contributing to this aim, these two interrelated studies were designed in order to explore, the perspectives of national and European level stakeholders, about the current situation on employment re-integration strategies for PwCDs and the prospects regarding the successful implementation of such strategies. 

The specific issues, examined in the studies, emerged from previous results in PATHWAYS on the identification of the existing and effective strategies implemented across the European countries and the assessment of employment needs of PwCDs (manuscript in preparation). In particular, a database of employment needs and the corresponding relevant strategies was set up. Based on the recommended available and effective strategies derived from the previous PATHWAYS studies, an interview protocol and a questionnaire were developed (see Section 2.3).

Data were collected from relevant national and European stakeholders through interviews and questionnaires, respectively. The data were also analysed from the perspective of four European welfare models. Each of these models, namely the Scandinavian, the Continental, the Mediterranean and the Post-Communist model, was represented by at least one country, involved in the national-level survey (see Table 1). 

### 2.2. Participants

With respect to the 1st study (“national-level survey”), convenience sampling was used as the main sampling procedure. Specifically, fifty-eight national stakeholders—including policy makers, experts/professionals in the field of employment re-integration of PwCDs and employers in the private sector—located in eight European countries participated. The total sample (*n* = 58) consisted of 14 policy makers (24.1%, 21 experts/professionals (36.2%) and 23 employers (39.7%) (see Table 1).

With respect to the 2nd study (“European-level survey”), European organizations with expertise on employment and health issues were invited to complete an online questionnaire. The main selection criterion was their active involvement in the work re-integration of PwCDs, through policy development and/or implementation. A total of 20 respondents/representatives of European organizations participated, representing seven European public organizations and 13 non-profit organizations: employed staff (*n* = 11), members of the board of Directors (*n* = 5), external collaborators (*n* = 2), an executive director and a junior researcher.

### 2.3. Material

For the aims of the national-level survey, a structured interview protocol was developed, consisting of two main sections:

Section A: Views on leadership and implementation of relevant policies. The participants were asked to specify the perceived availability of specific strategies, facilitating the employment re-integration of PwCDs. For each statement, three answer options were provided: 1. Yes, 2. No, and 3. “I don’t know”.

Section B: Recommended strategies in the labour market. Fourteen policy recommendations, targeting the re-integration into work and sustainable employment of PwCDs, were evaluated. The recommendations were rated in terms of perceived importance on a three-point Likert scale (1. not at all important to 3. very important) and with respect to perceived implementation (1. not at all implemented to 3. fully implemented). The possibility to answer “I don’t know” was also available. For each recommendation, 11 factors, reflecting potential barriers (e.g., inadequate legislation, lack of resources, lack of awareness, lack of expertise), were presented and the participants were asked to check up to five which, according to their view, hinder its effective implementation. 

Correspondingly, an online questionnaire was developed, in line with the aims of the European-level survey. The questionnaire was structured in three sections: 

Section A: General information about the respondent stakeholder including demographic information about their current position and their organization (e.g., name, type, governmental level). 

Section B: Policy directions, legislation and policy implementation. This section included statements referring to the availability of specific policy directions and legislation (seven statements) and policy implementation (six statements), targeting to the employment re-integration of PwCDs, at a European level. A three-point Likert scale was used. The possibility to answer “I don’t know” was also available. Participants were able to further comment their choice. 

Section C: Policy recommendations. The participants were asked to evaluate the importance of 19 policy recommendations, rated on a four-point Likert scale. The participants could comment on the recommendations and indicate any other policy recommendation, they considered important. 

### 2.4. Data Collection 

Eight PATHWAYS Partners from seven European countries (Austria, Czech Republic, Italy, Greece, Norway, Poland, Slovenia), representing the four European welfare models, identified national stakeholders and invited them to participate. All Partners conducted at least eight interviews with two policy makers, three professionals and three employers from each country. The 58 interviews were conducted from May to June 2017. Each Partner sent the extracted interviews’ data to OR and DP, for further processing and analysis. For the data collection of the European-level survey a list of 60 potential participating European organizations with active involvement/contribution to employment re-integration policy development and/or implementation was developed by Pathways Partners. To approach participants from different regions in Europe, an online version of the questionnaire was developed and implemented using Google forms. Contact persons from each European organization received an email invitation, including information on the PATHWAYS Project, the purpose of the study and the survey link. Non-respondents were sent an average of four reminder emails. Twenty organizations’ representatives completed the online questionnaire (response rate 33.3%), three refused to participate in the online survey, due to lack of time, while the remaining did not respond. The data were collected from March to May of 2017.

### 2.5. Data Analysis

Descriptive statistics were used to present the views of both national and European stakeholders on the issues examined in all sections of the interview protocol and the online questionnaire, respectively. Regarding the data from the national-level interviews, separate analyses were carried out for the total sample and the sub-groups, concerning the different types of stakeholders and the European welfare models, as well. In particular, Fisher exact tests were performed to examine whether there was a significant difference among the three types of national stakeholders or the four welfare models as regards responses on the 10 statements of Section A of the interview. For the items of Section B of the interview, referring to the importance and the level of implementation of the policy recommendations, median scores for the total sample and the sub-groups of the participants were calculated. The “I don’t know” responses were not included in such calculations. Kruskal-Wallis H tests were conducted to assess if there was a significant difference between the types of stakeholders or the welfare models regarding responses on both the importance and the implementation level of the recommendations. In addition, responses of the national and European participants to open-ended questions were categorized by Olga Roka. Results were examined and confirmed by Anastasia Vlachou and Panayiota Stavroussi. 

## 3. Results

### 3.1. National Level Survey

#### 3.1.1. Availability of Employment Re-Integration Policies at National Level

According to national stakeholders, most of the employment re-integration strategies were perceived as not being sufficiently available (see Table 2). The vast majority of participants reported lack of effective co-ordination in the implementation of policies for work re-integration, at both national (statement 5) and local levels (statement 6), (*n* = 45; 77.6% and *n* = 43; 74.1%, respectively), as well as non-adequate existing National legislation, either to reduce unemployment (statement 2) or to promote employment re-integration (statement 3), (*n* = 40; 69.0% and *n* = 39; 67.2% respectively). In contrast, the strategies for supporting the implementation of national policies for work re-integration by specialists (statement 8) obtained the highest rate of positive responses (*n* = 23; 39.7%). Interestingly, almost half of the participants (*n* = 25; 43.1%) were not aware, whether specific measures were set out on a national level for the evaluation of work re-integration policies.

A statistically significant difference was found in the responses of the different types of national stakeholders concerning the effective coordination of the implementation of policies (statement 6). Policy makers’ and professionals’ responses differed significantly (*p* = 0.01), as professionals responded more negatively regarding coordination of policies.

With respect to the European welfare models, significant differences, between the responses of participants from the Scandinavian and Post-Communist models were observed regarding statements 1 (*p* = 0.021), 5 (*p* = 0.042) and 7 (*p* = 0.017). More negative responses were given by the participants from the Post-Communist model, in the three statements. Also, a significant difference was revealed, between the responses of the participants from the Continental and Mediterranean model, regarding statement 8 (*p* = 0.049), as more positive responses were given by the participants from the Continental model (see Table 2 for the actual statements).

#### 3.1.2. Importance and Level of Implementation of the Policy Recommendations at National Level

**Figure 1:**
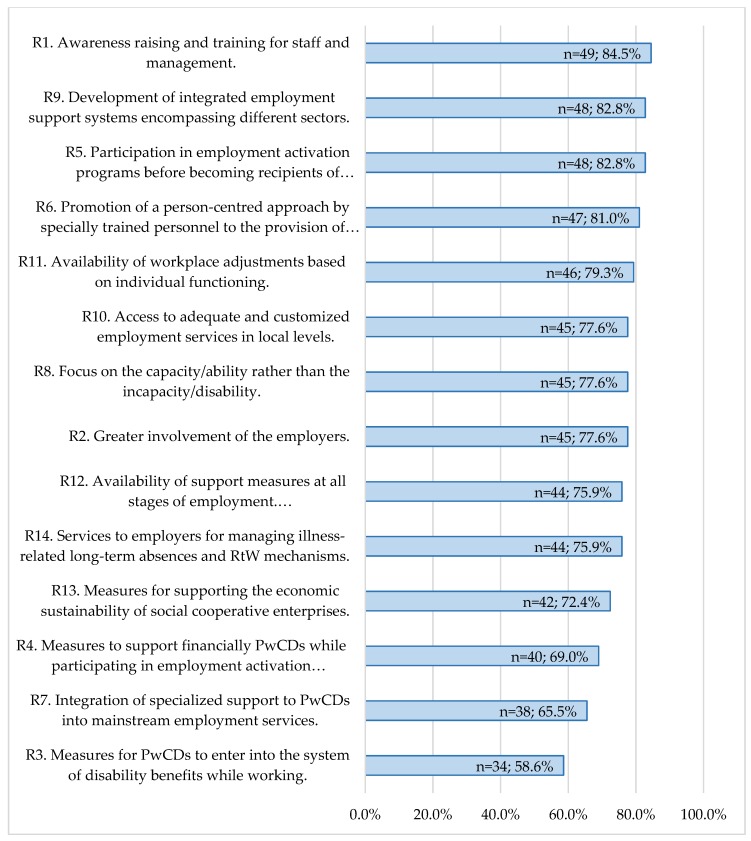
Recommendations’ ranking based on “very important” response rates of the national stakeholders.

All proposed recommendations were considered “very important” from at least half of the participants (see Figure 1). Recommendation 1 (awareness raising and training for staff and management to better understand the needs of PwCDs) was the most important followed by Recommendation 9 and Recommendation 5. Recommendation 3 had the lowest “very important” response rate (*n* = 34; 58.6%) (see Figure 1).

Analysing the views of the different types of national stakeholders on perceived importance of the suggested recommendations it was found that: a. Recommendations 1,6 and 10 were considered very important, by the vast majority of professionals (*n* = 20; 95.2%); b. Recommendation 1 had the highest “very important” response rate by policy makers (*n* = 12; 85.7%), while employers gave their most “very important” responses (*n* = 20; 85.7%) to the need of placing support measures (i.e., job coaching, mentoring, counselling) for PwCDs at all stages of employment, both in finding and maintaining a job (Recommendation 12); c. Recommendation 4, was not considered as “very important”. Recommendation 4 was found to be significantly more important for professionals than for policy makers (*p* = 0.41, *r* = 0.41).

**Figure 2:**
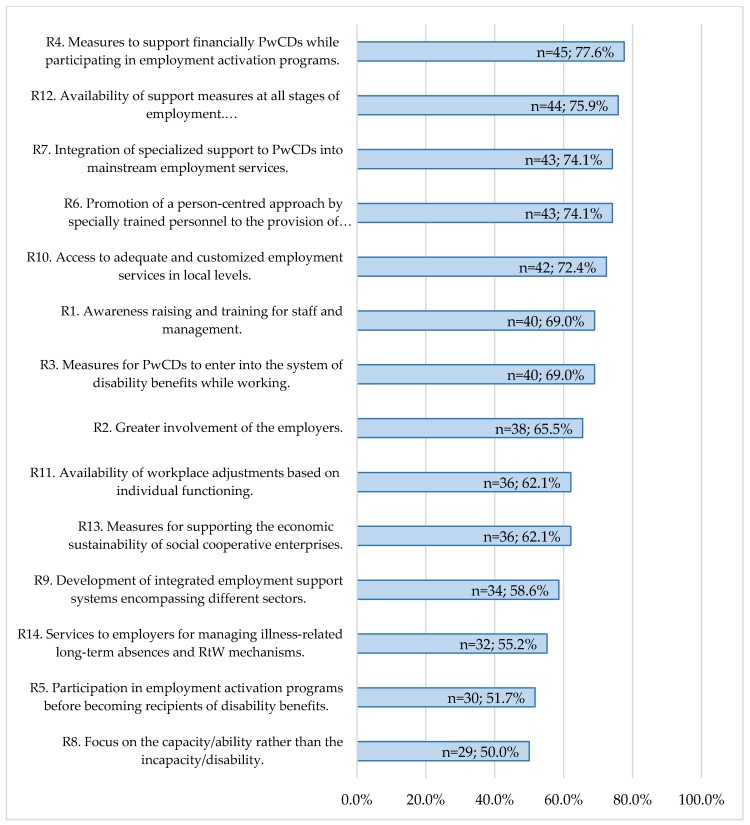
Recommendations “partially” and “fully implemented” according to national stakeholders’ views.

Regarding the level of implementation (see Figure 2), the highest value was recorded for Recommendation 4 (partially implemented: *n* = 41; 70.7% and fully implemented: *n* = 4; 6.9%), while the lowest value was provided for Recommendation 8 (partially implemented: *n* = 26; 44.8% and fully implemented: *n* = 3; 5.2%). 

The level of implementation of Recommendation 12 was associated with the different welfare models (*p* = 0.02). The availability of support measures—i.e., job coaching, mentoring, counselling—at all stages of employment was ranked as being less implemented by the respondents of the Mediterranean model, compared to those of the Continental (*p* = 0.027, *r* = 0.482). Further, Recommendation 5 seems to be implemented in a greater extent in the Scandinavian and the Continental model with mean ranks 31.5 and 27.9, respectively, compared to the Mediterranean with a mean rank of 17.73, even if the difference was not significant. 

#### 3.1.3. Barriers for Effective Implementation of the Policy Recommendations

**Figure 3:**
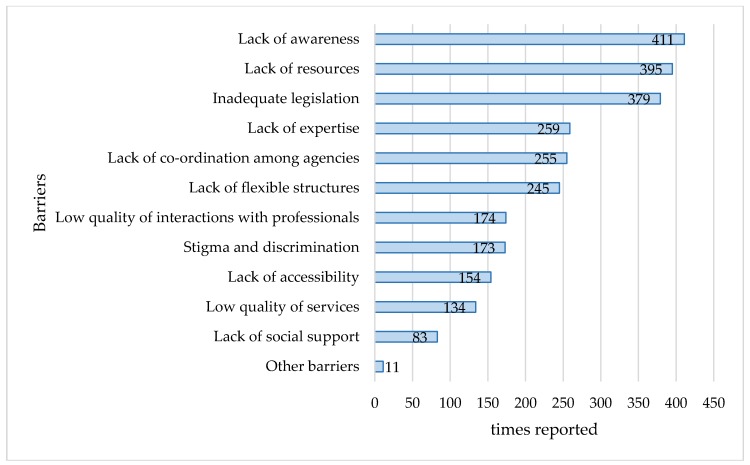
Most frequently referred barriers to the implementation of the recommendations.

As shown in Figure 3, the most frequently reported barriers were “lack of awareness”, followed by “lack of resources”, while “lack of social support” was the least frequently referred ones. Three additional types of barriers emerged from the open questions of the semi-structured interview: “requirements of the current labour market” (i.e., strong focus on performance in the companies, competition), “lack of incentives” (i.e., limited incentives for both employers and potential employees with chronic diseases) and “lack of clear procedures and effective measures”.

### 3.2. European-Level Survey

European-level participants evaluated 13 statements, regarding the effective conceptualization and implementation of strategies at a European level (see Table 3). 

Most of the policy and legislative, directions described at the first seven statements, were rated as moderately available (“to some extent”), from, at least, half of the participants. Exception to the above was the statement 5e with nine participants (45%) supporting its moderate availability. The highest “to some extent” response rate was found for statement 3 (*n* = 17; 85%), followed by the statement 5d (*n* = 16; 80%) (see Table 3). The highest rating, that is “to a great extent” response, was recorded for statement 5c, reported by 30% (*n* = 6) of the participants. In contrast, the 30% and 40% of the participants pointed out the absence of European directives for reducing unemployment (statement 6) and for facilitating the re-integration of PwCDs into the open labour market (statement 7).

Most strategies described at the last six statements of Table 3 were considered poorly implemented. In particular, “to a great extent” responses were reported by only two participants regarding statement 11 while statements 10 and 13 were rated as being implemented “to a great extent” by only one participant each. Two statements (12 and 13) were considered as being “to some extent” implemented by at least half of the participants, while statements 12 and 8 received the highest “not at all” response rates (*n* = 8, 40%). Interestingly, for almost half of the statements, participants’ responses were scattered across the different answer options. 

As far as the existence of specific outcome measures (statement 9) and the production of statistics (statement 10) for evaluating work re-integration policies are concerned, many participants (45% and 35%, respectively) used the “I don’t know” option. Five participants (25%) reported lack of both outcome measures and statistics for the evaluation of re-integration strategies and almost one-third of the participants mentioned the moderate implementation of evaluations. These findings suggest either lack of knowledge, from the participants’ side, or lack of monitoring systems/mechanisms regarding the evaluation of the existing employment re-integration strategies at a European level.

**Figure 4:**
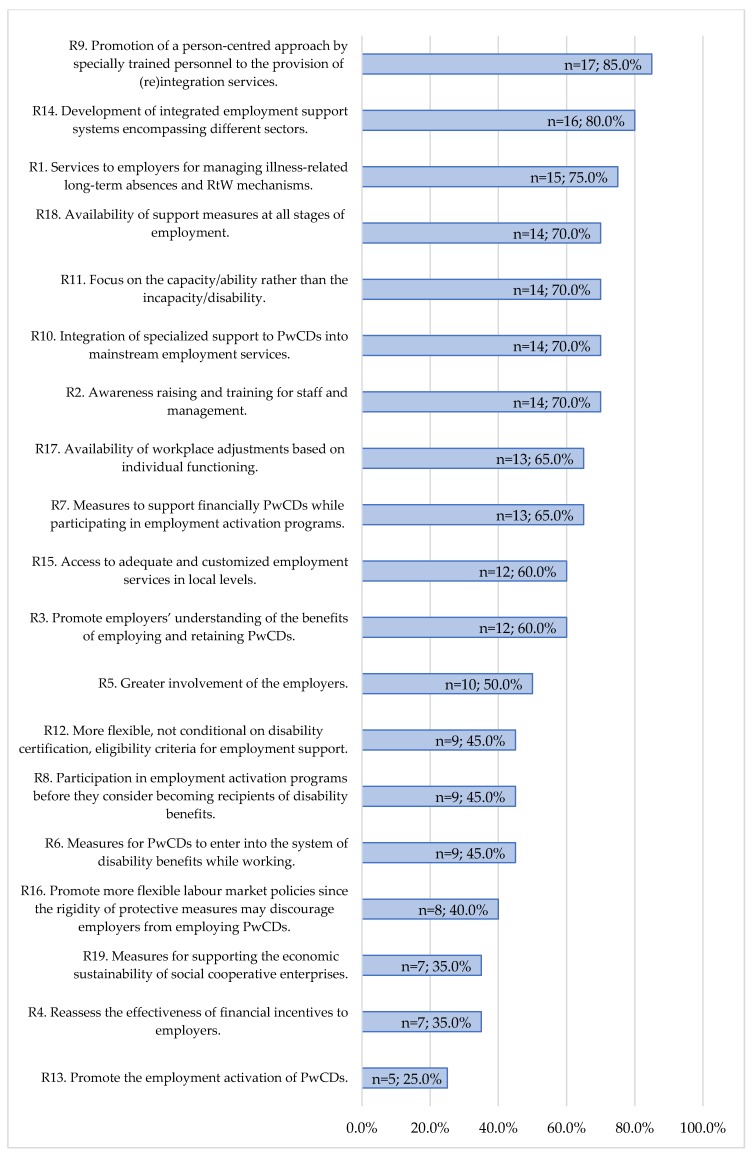
Recommendations’ ranking based on “very important” response rates.

In addition, participants rated the importance of 19 policy recommendations. Figure 4 shows the ranking of “very important” response rates. 12 out of 19 recommendations were considered as “very important” by at least half of the participants. Recommendation 9 was the most important, according to 17 participants (85%), followed by recommendation 14, (*n* = 16, 80%). In contrast, the “emphasis on the design and implementation of policies, targeting exclusively the employment activation of persons with chronic diseases” (recommendation 13) had the lowest rate of importance: only 25% of participants considered it as “very important”.

Twelve participants provided comments on challenges and barriers for effective implementation of specific recommendations and employment re-integration strategies of PwCDs. The most referred challenges/barriers were lack of awareness, followed by stigma, discrimination and misconceptions. Other challenges included: lack of resources, difficulties in collaboration between different sectors/actors, lack of incentives for employers (i.e., small and medium-sized enterprises and for (potential) employees with chronic diseases, limited access to appropriate healthcare and treatment. One comment was related to existing differences across the EU Member States, regarding both the definitions of “disability” and the effects of chronic diseases on employment-related issues, hampering development and implementation of a common policy line across the EU.

Finally, some of the main policy recommendations proposed by the European-level stakeholders included: the greater involvement of employers’ organizations; income benefits to support PwCDs, while participating in employment activation programmes; measures to support the employment re-integration of PwCDs designed specifically for each patients’ category and in the context of an inclusive approach, rather than a “one size fits all” approach; person-driven approach to the provision of employment re-integration services; consistent implementation of the existing European strategies (policies, systems, services); application of a social (considering the impact of a healthcare intervention on the individual’s ability to work and the economic effect) rather than a health care/economic perspective (based on costs and benefits of the healthcare system) to promote the re-integration of PwCDs into work; and close cooperation among the European Commission, the EU Member States and their social partners, to clarify the rights of PwCDs, highlight successful workplace adjustments and re-integration actions.

## 4. Discussion

The aim of this paper was to explore the current situation of strategies targeting employment re-integration of PwCDs in Europe and across European countries, and the opportunities to promote their participation in the labour market, from the perspective of both national and European level stakeholders. In overall, the vast majority of national stakeholders reported lack of effective coordination in the implementation of policies for work re-integration of PwCDs at both national and local levels. They considered the existing national legislation as inadequate to reduce the unemployment and to promote the employment re-integration of PwCDs. Raising awareness and training of service staff and human resources (HR) managers revealed as the most important strategy for promoting the labour-market participation of persons with chronic diseases. The provision of disability benefits to PwCDs, while working had the lowest rate of importance, but also the highest level of implementation, according to national stakeholders’ views. 

At the European-level, the availability of explicit provisions within EU policies to promote equality was supported by the majority of European-level participants. However, most of them considered that the reduction of unemployment and the employment re-integration of persons with chronic diseases were priorities “to some extent”, while the availability of the corresponding EU directives was questioned. A person-centred approach in providing re-integration services was indicated as the most important strategy. In contrast, the design and implementation of employment activation policies specifically for PwCDs was pointed out as the least important.

Our studies revealed that despite the “steps” made to protect the rights on employment of PwCDs as part of the broader group of persons with disabilities at a European level and across EU member states, the employment-related challenges faced by PwCDs in working age still remain unaddressed. Both national and European stakeholders reported that the existing national legislation and the European directives for reducing unemployment and promoting the re-integration into work are inadequate, while the co-ordination for the implementation of employment re-integration policies was evaluated as ineffective at multiple levels (i.e., local, national and European). These findings support previous arguments according to which the existing policies and legislative frameworks (e.g., anti-discrimination Acts, the UN Convention on the Rights of Persons with Disabilities) are not sufficient to enable the participation in the workforce, mainly due to challenges in transforming right-based policies into practices and establishing monitoring and evaluation systems for their effective implementation [17,18,24]. 

Other scholars have argued that the right-based policies and the development of inclusive labour markets impinge upon the current economic circumstances which impose cost limitations and labour force reductions for ensuring competitiveness in the global labour market [15,25]. This means that people who acquire or retain their jobs are those who can surpass the expectations of employers, in terms of qualifications, productivity and demands, i.e., those who at least meet the “able-body” norm.

### 4.1. Importance and Implementation of Policy Recommendations

With regard to the policy recommendations, the participants from both studies reported clearly the high importance of the majority of the strategies suggested. National and European stakeholders ranked among the most important recommendations the necessity of policy provisions to focus on the capacity/ability rather than the incapacity/disability of persons with chronic diseases to work and the need for developing integrated employment support systems for PwCDs in different sectors. The high priority given to the above recommendations highlights the need to abandon the medical approach to disability in order to promote the employment of persons with chronic diseases, while at the same time indicates that the adoption of a more holistic approach to the provision of support can assist people to overcome the barriers at multiple areas of their lives. As previous studies have indicated [26,27,28,29,30], employment strategies combining the provision of different types of support to persons with chronic diseases (e.g., support from professionals with different backgrounds in multidisciplinary interventions; mental health care incorporated in employment services for those with mental health issues), contributed decisively to the acquirement and/or maintenance of employment. 

Furthermore, the results showed that the economic dimension, reflected at certain recommendations related to economic support measures, influence the views of both national and European level participants. For instance, policies reflecting the provision of financial support or disability benefits to PwCDs while working received less support by the participants. The recommendation referring to the necessity of encouraging PwCDs to participate in employment activation programmes before receiving disability benefits was considered by the national stakeholders as very important but it was not considered so important by the European level stakeholders. At the same time, for many recommendations, the level of perceived importance was in inverse proportion to the level of their implementation and vice versa. This could be interpreted as an indication that, at an institutional level, “protective” financial incentives ensure an adequate level of financial gain for PwCDs seeking to enter and remain in the labour market and avoid the risk of poverty and material deprivation. From another perspective, providing financial/disability benefits could reproduce beliefs related to the limited productivity and capacity, while it may turn (potential) employees with chronic diseases into a cheap workforce [15].

Examining the barriers to the effective implementation of the policy recommendations, the results highlighted the lack of awareness as one of the most important challenges. While various national interventions have been implemented across the European countries for raising awareness regarding the employment of persons with chronic diseases [3], still the effectiveness of such initiatives in practice are not clear. Interestingly, lack of awareness was indicated as a barrier not only for the implementation of the recommendations referred to the employers and the workplace (e.g., the provision of reasonable accommodations), but also for those aiming at the activation of PwCDs. This may imply that information regarding the procedures and existing strategies, which act as incentives for persons with chronic diseases to return to work or to maintain employment, do not sufficiently reach the persons concerned. From the employers’ side, the implementation of practices increasing the opportunities for hiring and retaining persons with chronic diseases—such as providing internships and applying a diversity management policy [31,32]—and the commitment to comply with legal obligations presuppose and incorporate the provision of information and the raising of awareness about employment and health issues [33].

### 4.2. Employment (Re)Integration Strategies in the Context of the EU Welfare Models

The results showed that the current situation regarding the employment re-integration of PwCDs is not homogeneous throughout Europe. The significant differences found between participants from the Scandinavian and the Post-Communist model captured the diverse approaches on employment re-integration within the European context. In particular, participants from Norway (Scandinavian model) reported the availability of strategies, including the effective co-ordination in policy implementation and the determination of outcome measures for evaluating the employment re-integration policies at national level, which received the fewest positive response rates not only from participants belonging to the Post-communist model but also from the total sample of national respondents. These findings account for the policy framework adopted by Norway, which maintains a balance between compensation and activation measures [34] and provides valuable incentives to both employers and employees being at risk of sick leave or the exclusion of the labour market due to health issues. Following a holistic and preventative approach, such a framework determines specific procedures for achieving sustainable employment for PwCDs, regardless of being recognized as persons with disabilities and it is transferred into practice through the active involvement of relevant stakeholders (e.g., state social security institution, employers and service providers) [13]. Not surprisingly, the importance of the recommendation related to the provision of more services to employers for managing long-term absences and return-to work mechanisms was considered to be low by the participants belonging to the Scandinavian model compared to those of the other three models. This difference could be interpreted by the effectiveness of the already existing strategies promoting job retention, such as the part time sick leaves [35]. 

In addition, the differences found between the responses of the participants from the Mediterranean and both the Scandinavian and Continental models indicated the dominance of a protective—compensate orientation. Strategies aiming at entering into the labour market and maintaining employment are less-developed and target mainly to those with a degree of recognised disability [13]. This fact could explain the poor availability of support by specialists for implementing the national re-integration to work policies and also the lack of adequate measures focusing on the employment re-integration of PwCDs without a recognised disability. Similarly, the participants from the Mediterranean model highlighted the need for a paradigm shift mentioning (a) the high importance for policies focusing on the work ability/capacity and the provision of additional services to employers for facilitating the job retention of employees with chronic diseases and (b) the limited implementation of strategies activating potential employees to enter into the labour market and supporting them during their work trajectories. 

### 4.3. Contribution of the Study and Limitations

Although the promotion of the employment of persons with chronic diseases is a key issue in the European policy agenda, studies in this field are relatively limited. The present two studies, is to the best of our knowledge the first research endeavour which adopts a “bottom-up” approach and depicts the current situation of strategies targeting specifically to the employment re-integration and inclusion of PwCDs, at a European level and across European countries representing different welfare models. The evidence presented contribute significantly to the knowledge in this field by exploring the perspectives of different types of national and European stakeholders (e.g., policy makers, service providers, employers and organizations) and providing meaningful insights regarding the development and implementation of more inclusive policies and practices. At this point, however, it is important to acknowledge that the use of a convenience sampling method creates limitations, in terms of representativeness while caution is required in interpreting and generalizing the results due to the small sample sizes. In this context, the quantitative nature of the studies limits even further the inferences leading to the need for conducting further research in order to confirm the effect of the results presented at this paper. In fact, the complexity and multiplicity of the issues involved demand extensive research with larger samples and mixed research methodologies. 

## 5. Conclusions

Existing employment re-integration policies and practices were considered as not being sufficiently available and implemented at both European level and across European countries represented in the study. In addition, responses of national stakeholder revealed a relatively poor implementation of policy recommendations, despite these being perceived as highly important by both national and European participants. However, the differences across the European welfare models revealed not only the gap existing among the European countries in terms of available and implemented employment re-integration policies and strategies, but also the possibilities and the need to put into practice a common European framework to prevent the unemployment and the exclusion from the labour market of persons with chronic diseases. 

We therefore recommend that relevant policies at European level should be directed at (a) meaningful and consistent cooperation among the EU, the governments and the social partners; (b) the dissemination and the transfer of effective employment re-integration strategies and good practices, adapted to the conditions of each country; (c) the establishment of monitoring systems and evaluating measures as bases for promoting evidence-based strategies. 

Furthermore, the results clearly showed that appropriate support to employers’ needs should be accompanied with interventions for awareness raising as well as services for managing the long-term sick leaves and the return to work process. Respectively, the financial benefits to persons with chronic diseases should be used as an incentive for participating in the workforce, rather than a compensation for those remaining inactive. However, this perspective presupposes the provision of adequate measures for facilitating and assisting persons with chronic diseases to re-integrate into work, including the adoption of a person-centreed and individualized approach, in which the particular person with a chronic disease plays an essential role, and the provision of services incorporating supports in different sectors.

## Figures and Tables

**Table 1:** Sample characteristics.

European Welfare Model	European Countries	Type of Participants			
		Policy Makers (n)	Professionals (n)	Employers (n)	Total (n)
Scandinavian	Norway	2	3	3	8
Continental	Austria	2	3	4	9
	Slovenia	2	3	3	8
Mediterranean	Greece	2	3	4	9
	Italy	2	3	3	8
Post-Communist	Czech Republic	2	3	3	8
	Poland	2	3	3	8
		14	21	23	58

**Table 2:** Views on the availability of work re-integration strategies by country (*n*, %).

Statements	Austria				Czech Republic				Greece						Italy							
	Yes		No		Yes		No		Yes			No			Yes				No			
1. Unemployment reduction among PwCDs is currently very high on the National agenda.	4	44.4%	4	44.4%	1	12.5%	7	87.5%	0		0.0%	6		66.7%	4		50.0%		3	37.5%		
2. The existing National legislation for reducing unemployment among PwCDs in the open labour market is adequate.	1	11.1%	7	77.8%	2	25.0%	4	50.0%	1		11.1%	5		55.6%	1		12.5%		6	75.0%		
3. The existing National legislation for re-integrating PwCDs in the open labour market is adequate.	1	11.1%	7	77.8%	1	12.5%	5	62.5%	0		0.0%	5		55.6%	1		12.5%		6	75.0%		
4. Developing strategies for re-integrating PwCDs in the open labour market is a high priority on the National agenda.	3	33.3%	5	55.6%	0	0.0%	7	87.5%	1		11.1%	5		55.6%	2		25.0%		5	62.5%		
5. The implementation of policies for re-integrating to work PwCDs is effectively coordinated on national level.	1	11.1%	7	77.8%	0	0.0%	5	62.5%	0		0.0%	8		88.9%	1		12.5%		6	75.0%		
6. The implementation of policies for re-integrating to work PwCDs is effectively coordinated on local level.	0	0.0%	6	66.7%	1	12.5%	5	62.5%	0		0.0%	7		77.8%	1		12.5%		6	75.0%		
7. At National level, specific outcome measures have been set for the evaluation of policies targeting re-integration to work of PwCDs.	1	11.1%	1	11.1%	0	0.0%	5	62.5%	0		0.0%	2		22.2%	2		25.0%		4	50.0%		
8. The implementation of national policies for re-integrating to work PwCDs is supported by specialists in the area of work integration.	6	66.7%	0	0.0%	1	12.5%	4	50.0%	1		11.1%	4		44.4%	3		37.5%		3	37.5%		
9. Service providers are well informed about the rights of PwCDs concerning their re-integration to work.	3	33.3%	3	33.3%	1	12.5%	5	62.5%	1		11.1%	4		44.4%	1		12.5%		4	50.0%		
10. Service providers are well informed about the available services supporting re-integration to work of PwCDs.	2	22.2%	5	55.6%	1	12.5%	6	75.0%	0		0.0%	3		33.3%	1		12.5%		5	62.5%		
**Statements**	**Norway**				**Poland**				**Slovenia**						**Total**							
	**Yes**		**No**		**Yes**		**No**		**Yes**				**No**		**Yes**			**No**			**I don’t Know**	
1. Unemployment reduction among PwCDs is currently very high on the National agenda.	5	62.5%	3	37.5%	1	12.5%	7	87.5%	1	12.5%			6	75.0%	16	27.6%		36	62.1%		6	10.3%
2. The existing National legislation for reducing unemployment among PwCDs in the open labour market is adequate.	2	25.0%	6	75.0%	0	0.0%	7	87.5%	3	37.5%			5	62.5%	10	17.2%		40	69.0%		8	13.8%
3. The existing National legislation for re-integrating PwCDs in the open labour market is adequate.	2	25.0%	5	62.5%	0	0.0%	7	87.5%	3	37.5%			4	50.0%	8	13.8%		39	67.2%		11	19.0%
4. Developing strategies for re-integrating PwCDs in the open labour market is a high priority on the National agenda.	4	50.0%	4	50.0%	2	25.0%	6	75.0%	0	0.0%			5	62.5%	12	20.7%		37	63.8%		9	15.5%
5. The implementation of policies for re-integrating to work PwCDs is effectively coordinated on national level.	3	37.5%	5	62.5%	0	0.0%	8	100.0%	2	25.0%			6	75.0%	7	12.1%		45	77.6%		6	10.3%
6. The implementation of policies for re-integrating to work PwCDs is effectively coordinated on local level.	3	37.5%	5	62.5%	1	12.5%	7	87.5%	1	12.5%			7	87.5%	7	12.1%		43	74.1%		8	13.8%
7. At the national level, specific outcome measures have been set for the evaluation of policies targeting re-integration to work of PwCDs.	4	50.0%	2	25.0%	1	12.5%	7	87.5%	1	12.5%			3	37.5%	9	15.5%		24	41.4%		25	43.1%
8. The implementation of national policies for re-integrating to work PwCDs is supported by specialists in the area of work integration.	4	50.0%	2	25.0%	4	57.1%	2	28.6%	4	50.0%			3	37.5%	23	39.7%		18	31.0%		16	27.6%
9. Service providers are well informed about the rights of PwCDs concerning their re-integration to work.	5	62.5%	2	25.0%	3	42.9%	3	42.9%	4	50.0%			2	25.0%	18	31.0%		23	39.7%		16	27.6%
10. Service providers are well informed about the available services supporting re-integration to work of PwCDs.	4	50.0%	2	25.0%	3	42.9%	3	42.9%	3	37.5%			0	0.0%	14	24.1%		24	41.4%		19	32.8%

**Table 3:** Views of European stakeholders on the conceptualization and implementation of employment re-integration policies and practices.

Statements	1 Not at All	2 To Some Extent	3 To a Great Extent	4 I Don’t Know
*Policy directives and legislation*				
1. Unemployment reduction among PwCDs is currently very high on the EU agenda.	2 (10.0%)	14 (70.0%)	3 (15.0%)	1 (5.0%)
2. Unemployment reduction among PwCDs is currently very high on the agenda of EU member states.	3 (15.0%)	12 (60.0%)	3 (15.0%)	2 (10.0%)
3. Policies and strategies for re-integrating PwCDs in the open labour market are a high priority on the EU agenda.	1 (5.0%)	17 (85.0%)	1 (5.0%)	1 (5.0%)
4. Policies and strategies for re-integrating PwCDs in the open labour market are a high priority on the agenda of EU member states.	1 (5.0%)	14 (70.0%)	3 (15.0%)	2 (10.0%)
5. EU policies related to the following areas make explicit provisions for PwCDs:				
a. Health services	2 (10.0%)	12 (60.0%)	3 (15.0%)	3 (15.0%)
b. Economic development	3 (15.0%)	12 (60.0%)	0 (0.0%)	5 (25.0%)
c. Equality	2 (10.0%)	11 (55.0%)	6(30.0%)	1 (5.0%)
d. Employment	0 (0.0%)	16 (80.0%)	3 (15.0%)	1 (5.0%)
e. Education	2 (10.0%)	9 (45.0%)	2 (10.0%)	7 (35.0%)
6. European directives for reducing unemployment among PwCDs in the open labour market are adequate.	6 (30.0%)	12 (60.0%)	0 (0.0%)	2 (10.0%)
7. European directives for re-integrating PwCDs in the open labour market are adequate.	8 (40.0%)	11 (55.0%)	0 (0.0%)	1 (5.0%)
*Policy implementation*				
8. The implementation of EU policies for re-integrating at work PwCDs is effectively coordinated among agencies within each EU member state.	8 (40.0%)	5 (25.0%)	0 (0.0%)	7 (35.0%)
9. At EU level, specific outcome measures have been set for evaluating policies for re-integrating at work PwCDs.	5 (25.0%)	6 (30.0%)	0 (0.0%)	9 (45.0%)
10. At EU level, statistics are produced for evaluating policies for re-integrating at work PwCDs.	5 (25.0%)	7 (35.0%)	1 (5.0%)	7 (35.0%)
11. The implementation of EU policies for re-integrating PwCDs is supported by specialists in the area of work integration at EU member state organizations.	3 (15.0%)	9 (45.0%)	2 (10.0%)	6 (30.0%)
12. PwCDs are well informed about their rights and the available services supporting their re-integration at work.	8 (40.0%)	10 (50.0%)	0 (0.0%)	2 (10.0%)
13. Service providers are well informed about the rights of PwCDs and the available services supporting their re-integration at work.	5 (25.0%)	13 (65.0%)	1 (5.0%)	1 (5.0%)
